# Transforming growth factor-β is involved in maintaining oocyte meiotic arrest by promoting natriuretic peptide type C expression in mouse granulosa cells

**DOI:** 10.1038/s41419-019-1797-5

**Published:** 2019-07-22

**Authors:** Jing Yang, Yu Zhang, Xiaoting Xu, Jia Li, Feifei Yuan, Shumin Bo, Jie Qiao, Guoliang Xia, Youqiang Su, Meijia Zhang

**Affiliations:** 1 State Key Laboratory for Agrobiotechnology, College of Biological Sciences, China Agricultural University, 100193 Beijing, China; 2 Capital University of Physical Education and Sports, 100191 Beijing, China; 3 Department of Obstetrics and Gynecology, Reproductive Medical Center, Peking University Third Hospital, 100191 Beijing, China; 4 State Key Laboratory of Reproductive Medicine, Nanjing Medical University, 211166 Nanjing, Jiangsu China

**Keywords:** Reproductive biology, Endocrinology

## Abstract

Natriuretic peptide type C (NPPC) secreted by mural granulosa cells (MGCs) maintains oocyte meiotic arrest via the activation of guanylyl cyclase-linked natriuretic peptide receptor 2 (NPR2). Here, we investigated the effect of transforming growth factor (TGF)-β on NPPC expression in MGCs and oocyte maturation. TGF-β ligands (TGFB1 and TGFB3, but not TGFB2) and receptors (TGFBR1 and TGFBR2) were predominantly expressed in MGCs. The activation of the follicle-stimulating hormone (FSH) receptor by FSH/equine chorionic gonadotropin (eCG) increased the levels of TGFB1, TGFBR2, and TGF-β downstream SMAD proteins in MGCs, which were decreased following the activation of the luteinizing hormone (LH) receptor by human chorionic gonadotropin (hCG). TGF-β significantly increased the gene and protein levels of NPPC in cultured MGCs through SMAD3 binding to *Nppc* promoter regions. In the presence of FSH, TGF-β further increased NPPC levels and inhibited oocyte meiotic resumption of cumulus-oocyte complexes (COCs). Moreover, *Tgfbr2*-specific depletion in granulosa cells using *Fshr-Cre* mice reduced NPPC mRNA and protein levels, resulting in the weak maintenance of oocyte meiotic arrest within large antral follicles. *Tgfbr2* depletion also impaired follicle development, ovulation, and female fertility. Taken together, TGF-β-promoted NPPC in MGCs is involved in maintaining oocyte meiotic arrest. FSH and LH could regulate NPPC levels in MGCs via TGF-β and then control the process of oocyte meiosis.

## Introduction

Mammalian oocyte meiosis begins in the fetal period but is arrested at the diplotene stage of the first meiotic prophase around the time of birth for a prolonged period^1,2^. Oocytes within preantral follicles do not possess the competence to progress through meiosis due to low levels of cell cycle regulatory proteins inherent in the oocyte^3^. When a fluid-filled follicle antrum forms, the arrested oocytes reach their full size and have the ability to resume meiosis^4,5^. However, natriuretic peptide type C (NPPC, also known as CNP), secreted by mural granulosa cells (MGCs), maintains oocyte meiotic arrest via its cognate receptor natriuretic peptide receptor 2 (NPR2) producing cyclic guanosine monophosphate (cGMP)^6–9^.

The activation of the follicle-stimulating hormone (FSH) receptor by FSH/equine chorionic gonadotropin (eCG, a glycoprotein hormone that primarily has FSH activity) leads to the formation of early antral follicles that eventually develop into preovulatory follicles^10^. During follicle development, the levels of NPPC/NPR2 in granulosa cells are increased to ensure oocyte meiotic arrest. The activation of the luteinizing hormone (LH) receptor by LH/human chorionic gonadotropin (hCG, a pregnancy hormone that exhibits LH activity) leads to a decrease in NPPC/NPR2 levels^11–14^, and NPR2 activity by dephosphorylation and a reduction in NPPC-binding affinity^15–17^. All of these factors reduce the production of cGMP, thereby relieving cGMP-mediated inhibition of meiotic arrest^7,8^. In vitro evidences suggest that the regulation of the expression of NPPC/NPR2 in granulosa cells by FSH and LH could be an indirect effect that is mediated by autocrine factors produced by granulosa cells. For example, previous studies showed that estradiol (E2) promotes *Nppc* and *Npr2* mRNA expression in cultured MGCs^14,18^. Given that FSH and LH affect E2 biosynthesis by specifically regulating the aromatizing enzyme system^19,20^, FSH and LH could regulate NPPC/NPR2 levels through the control of E2 production. However, aromatase deletion had no effect on oocyte maturation, and estrogen receptor (ER) knockout only had a partial effect on oocyte meiotic resumption^21,22^. Thus, other factors could also be involved in promoting the expression of NPPC/NPR2 for meiotic arrest^23^.

NPPC expression and secretion are increased by transforming growth factor β (TGF-β) in human and rat vascular smooth muscle cells and bovine aortic endothelial cells^24–26^. In mammals, the TGF-β family includes three ligands: TGFB1, TGFB2, and TGFB3. These ligands bind to their membrane receptors and trigger the serine/threonine protein kinase activity of type 2 receptor (TGFBR2) and type 1 receptor (TGFBR1), and then the activated receptor phosphorylates intracellular SMAD2 (Sma- and Mad-related protein 2) and SMAD3. Subsequently, phosphorylated SMAD2 and SMAD3 form a heteromeric complex with SMAD4 (co-SMAD, the central signaling component of the TGF-β superfamily)^27,28^. Ultimately, the complex translocates into the nucleus and regulates gene expression by binding to the target gene promoter region, termed an SMAD-binding element (SBE), and recruiting distinct transcription factors^29^.

The expression of TGF-β ligands and their receptors has been observed in mammalian ovaries from different species^30–32^. Accumulating evidence indicates that TGF-β participates in multiple ovary functions, including follicle growth^33^, granulosa cell proliferation^34–36^, and ovulation^37^. Moreover, TGFB1 had an inhibitory effect on the spontaneous meiotic resumption of porcine cumulus-oocyte complexes (COCs)^38^. Although *Smad4* depletion in granulosa cells decreases the mRNA levels of *Nppc* and *Npr2*, resulting in the weak maintenance of oocyte meiosis arrest^39^, its specific upstream signaling molecule(s) is unclear. Thus, we investigated the expression pattern of TGF-β ligands and receptors in mouse ovaries and their effects on NPPC/NPR2 expression and oocyte maturation.

## Results

### The expression pattern of TGF-β ligands and receptors in mouse ovary

**Fig. 1:**
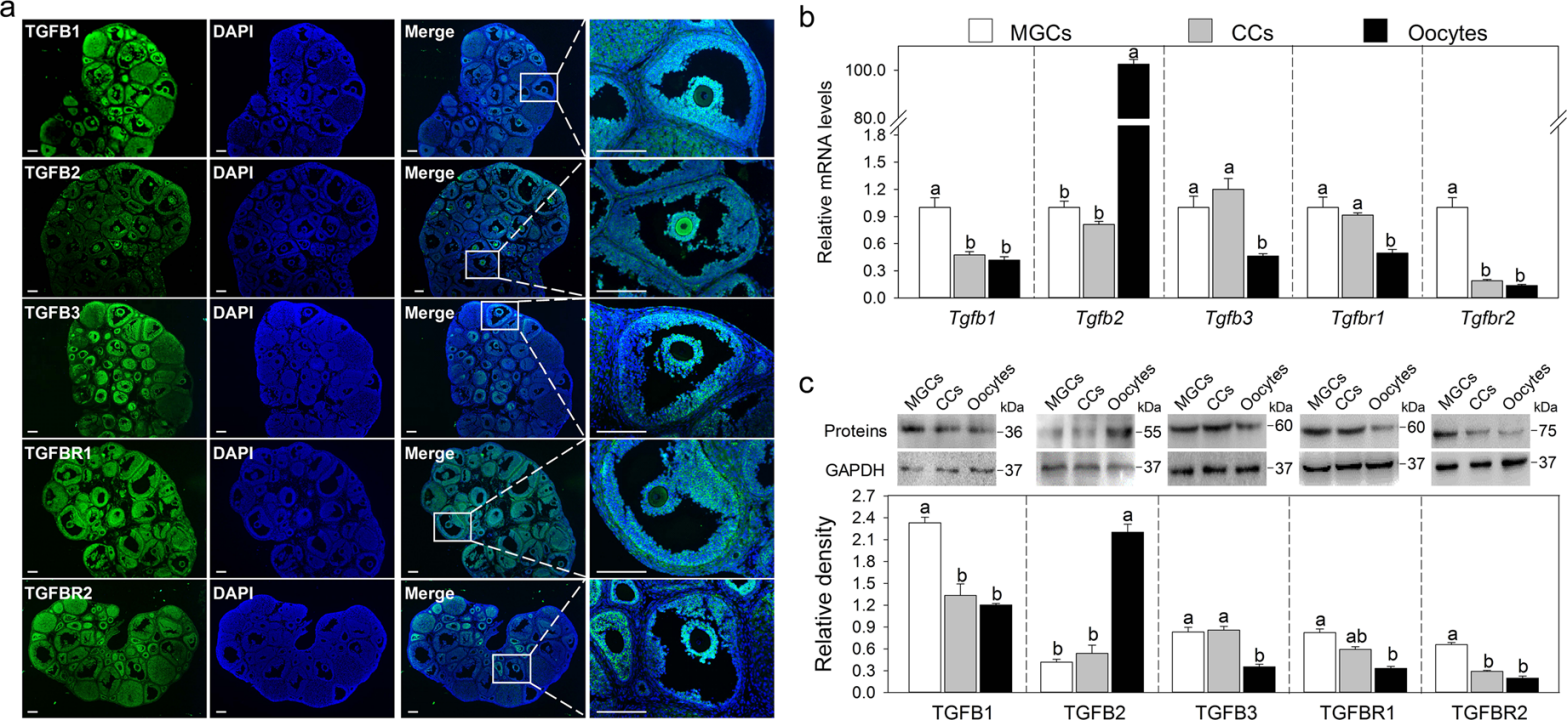
The expression pattern of TGF-β ligands and receptors in mouse ovaries. **a** Immunofluorescence analysis of TGFB1, TGFB2, TGFB3, TGFBR1, and TGFBR2 in the ovaries isolated from eCG-primed mice. The images are representative of three images captured. The small white boxes indicate the enlarged areas as shown in the following images. Scale bars: 200 μm. **b** Comparison of steady-state levels of *Tgfb1, Tgfb2, Tgfb3, Tgfbr1*, and *Tgfbr2* mRNA in MGCs, cumulus cells (CCs) and oocytes isolated from eCG-primed mice. **c** Representative western blotting of TGFB1, TGFB2, TGFB3, TGFBR1, and TGFBR2 in MGCs, CCs and, oocytes isolated from eCG-primed mice. GAPDH was used as a loading control. Bars indicate the mean ± SEM of three independent replicates. Values not indicated by the same letter are significantly different (*P* < 0.05)

We first detected the localization of TGF-β ligands and receptors in eCG-primed mouse ovaries by immunofluorescence. TGFB1, TGFB3, TGFBR1, and TGFBR2 were clearly detected in the cytoplasm of MGCs and cumulus cells, and TGFB1 and TGFBR1 staining were also weak in the oocyte cytoplasm (Fig. 1a). In contrast, TGFB2 was mainly located in the cytoplasm of oocytes and was slightly stained in the cytoplasm of MGCs and cumulus cells (Fig. 1a). Theca cells had a slight expression of these ligands and receptors (Fig. 1a).

Next, we compared the expression levels of TGF-β ligands and receptors in MGCs, cumulus cells, and oocytes. The mRNA and protein levels of TGFB1 and TGFBR2 in MGCs were higher than in cumulus cells and oocytes (Fig. 1b, c). TGFB3 and TGFBR1 levels were higher in both MGCs and cumulus cells compared with those in oocytes (Fig. 1b, c). TGFB2 mRNA and protein levels were dramatically higher in oocytes than in MGCs and cumulus cells, which are consistent with the immunofluorescence results (Fig. 1b, c). Collectively, TGFB1, TGFB3, TGFBR1, and TGFBR2 are mainly expressed in MGCs, and TGFB2 is mainly expressed in oocytes. We speculate that TGF-β plays a physiological role in these cells.

### Gonadotropins regulate the expression of TGF-β signaling molecules in MGCs in vivo

**Fig. 2:**
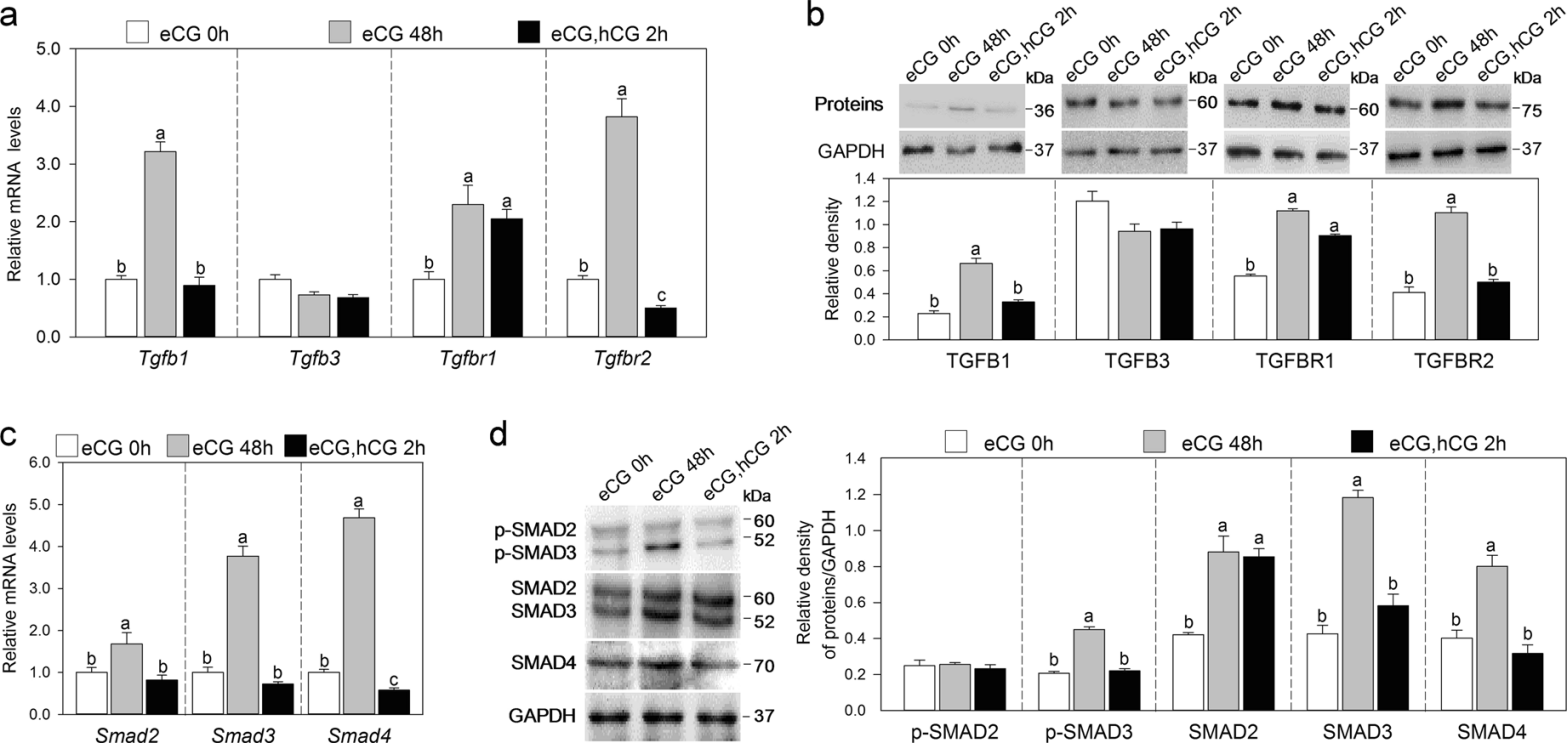
Gonadotropins regulate the expression of TGF-β signaling molecules in MGCs. MGCs were isolated from prepubertal mice, eCG-primed mice and eCG-primed mice followed by hCG as indicated in the figures. **a** Quantitative RT-PCR analysis of the gene expression of *Tgfb1, Tgfb3, Tgfbr1*, and *Tgfbr2* in MGCs. **b** Western blotting analysis of the protein levels of TGFB1, TGFB3, TGFBR1, and TGFBR2 in MGCs. **c** Quantitative RT-PCR analysis of the gene expression of *Smad2, Smad3*, and *Smad4* in MGCs. **d** Western blotting analysis of the protein levels of SMAD2/3, SMAD4, and phosphorylated SMAD2/3 (p-SMAD2/3) in MGCs. The blots shown are representative of three images captured in **b**, **d**. GAPDH was used as a loading control in **b**, **d**. Bars indicate the mean ± SEM of three independent replicates. Values not indicated by the same letter are significantly different (*P* < 0.05)

We studied the effect of eCG on the expression of TGF-β signaling molecules in MGCs during follicle development. Treatment with eCG significantly increased the mRNA levels of *Tgfb1, Tgfbr1*, and *Tgfbr2* in MGCs (Fig. 2a). Consistent with this, western blotting results showed that the protein levels of TGFB1, TGFBR1, and TGFBR2 were significantly increased (Fig. 2b). Furthermore, eCG significantly increased the mRNA and protein levels of SMAD2, SMAD3, and SMAD4 (Fig. 2c, d). The phosphorylation levels of SMAD3, but not SMAD2, were also increased (Fig. 2d). We next examined the expression change in TGF-β signaling molecules in MGCs during hCG-induced oocyte maturation. After hCG treatment, the mRNA and/or protein levels of TGFB1, TGFBR2, SMAD3, SMAD4, and phosphorylated SMAD3 were decreased to pre-eCG levels (Fig. 2a–d). Gonadotropins had no effect on the expression of TGFB2 in oocytes (Fig. S2a) and TGFB3 in MGCs (Fig. 2a, b), indicating that other factor(s) regulates their expression.

### FSH upregulates TGF-β signaling molecules in MGCs in vitro

**Fig. 3:**
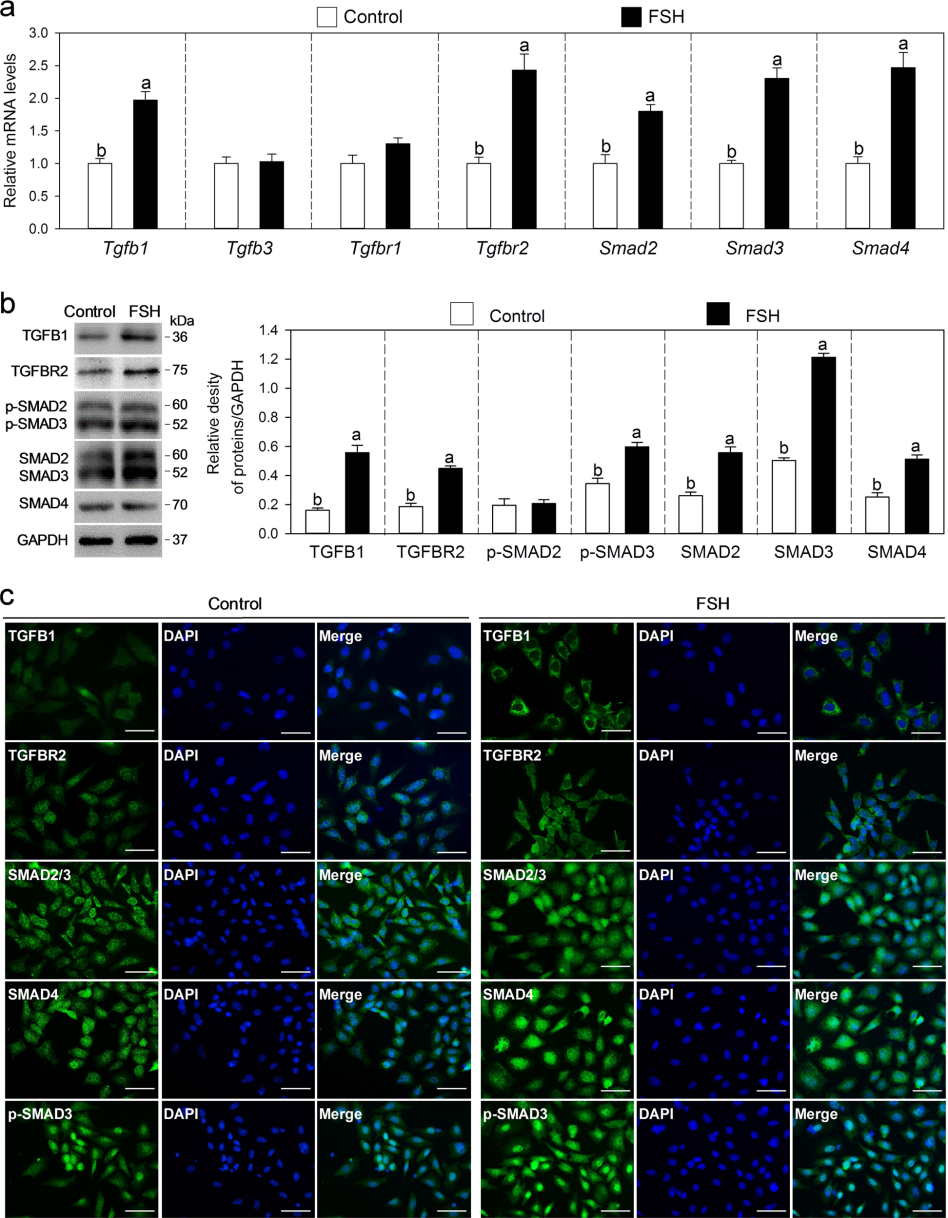
FSH upregulates TGF-β signaling molecules in MGCs. MGCs collected from prepubertal mice were cultured for 24 h in cell culture medium without or with 10 ng/ml FSH**. a** Effects of FSH on *Tgfb1, Tgfb3, Tgfbr1*, *Tgfbr2, Smad2, Smad3*, and *Smad4* mRNA levels in MGCs. **b** Effects of FSH on the protein levels of TGFB1, TGFBR2, SMAD2/3, SMAD4, and phosphorylated SMAD2/3 (p-SMAD2/3) in MGCs. The blots shown are representative of three images captured. GAPDH was used as a loading control. **c** Immunofluorescence analysis for TGFB1, TGFBR2, SMAD2/3, SMAD4, and phosphorylated SMAD3 (p-SMAD3) in MGCs. The images are representative of three images captured. Scale bars: 50 μm. Bars indicate the mean ± SEM of three independent replicates. Values not indicated by the same letter are significantly different (*P* < 0.05)

We studied the effect of FSH on the expression of TGF-β signaling molecules by culturing MGCs. FSH treatment significantly increased the mRNA levels of *Tgfb1*, *Tgfbr2*, *Smad2*, *Smad3*, and *Smad4* (Fig. 3a). Consistent with the results of gene expression, western blotting analyses revealed that an FSH significantly increased the protein levels of TGFB1, TGFBR2, SMAD2, SMAD3, SMAD4, and phosphorylated SMAD3 (Fig. 3b). Immunofluorescence results also showed that FSH treatment obviously increased the cytoplasm accumulation of TGFB1 and TGFBR2 and the nuclear accumulation of SMAD4 and phosphorylated SMAD3 (Fig. 3c). Thus, FSH upregulates TGF-β signaling molecules in MGCs and activates the TGF-β signaling pathway directly.

### TGF-β promotes NPPC expression and inhibits oocyte meiotic resumption

**Fig. 4:**
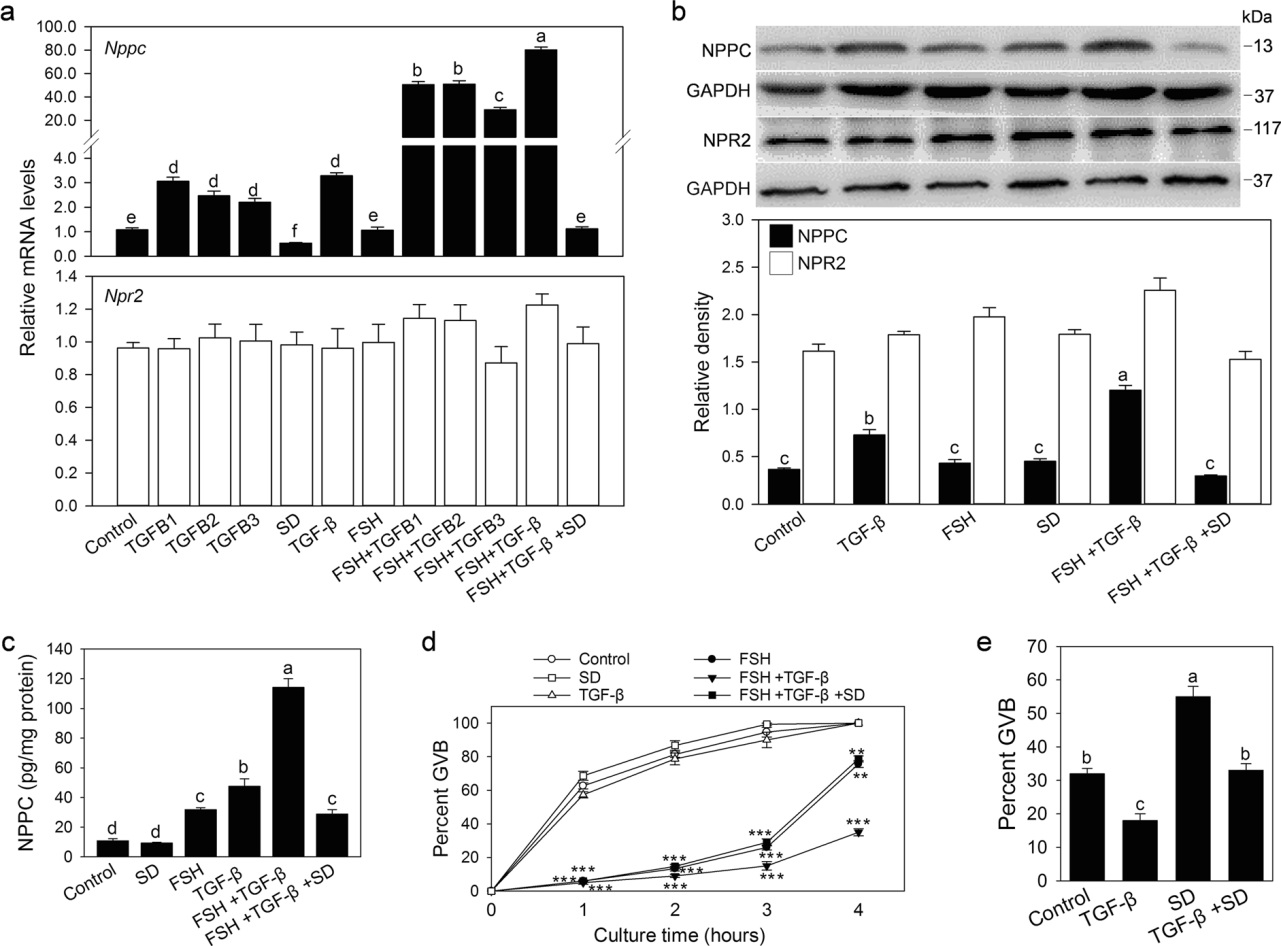
TGF-β promotes NPPC expression and maintains oocyte meiotic arrest. **a–c** MGCs were collected from prepubertal mice and cultured for 24 h in cell culture medium supplemented with 10 ng/ml TGFB1, 50 ng/ml TGFB2, 10 ng/ml TGFB3, TGF-β (including 10 ng/ml TGFB1, 50 ng/ml TGFB2, 10 ng/ml TGFB3), 1 μmol/l SD208 (SD), and/or 10 ng/ml FSH. NPPC and/or NPR2 mRNA and protein levels were detected by qRT-PCR (**a**), western blotting (**b**) and a luciferase immunoassay (**c**), respectively. The blots shown are representative of three images captured. GAPDH was used as a loading control. **d** Effect of TGF-β on oocyte meiotic resumption of COCs. MGCs were cultured in the medium supplemented with TGF-β, SD208, and/or FSH for 24 h, and then COCs isolated from eCG-primed mice were cocultured with MGCs for 1, 2, 3, and 4 h. *n* = 30 for each treatment across all three replicates. **P ˂ 0.01 and ***P ˂ 0.001 compared with corresponding control. **e** Effect of TGF-β on the meiotic resumption of oocytes within follicles. Large antral follicles isolated from eCG-primed mice were cultured in the medium supplemented with TGF-β, and/or SD208 for 4 h. *n* = 30 for each treatment across all three replicates. Bars indicate the mean ± SEM of three independent replicates. Values not indicated by the same letter are significantly different (*P* < 0.05)

We cultured MGCs to investigate the effect of TGF-β on NPPC expression. As a result, TGFB1, TGFB2, TGFB3, or the combination of these three TGF-β ligands (here as TGF-β) promoted the expression of *Nppc* mRNA (Fig. 4a). Western blotting and a luciferase immunoassay showed that TGF-β increased NPPC protein levels (Fig. 4b, c), which is consistent with qRT-PCR results. FSH, as reported previously^18^, had no effect on NPPC expression (Fig. 4a, b) and significantly enhanced TGF-β-induced NPPC expression (Fig. 4a–c). TGF-β-promoted NPPC was completely reversed by the TGF-β signaling inhibitor SD208 (Fig. 4a, b). These results indicate that TGF-β upregulates NPPC expression in MGCs. However, TGF-β had no effect on the NPR2 gene and protein expression in cultured MGCs (Fig. 4a, b).

To study whether TGF-β had an inhibitory effect on oocyte meiotic resumption by promoting NPPC expression, MGCs were cultured in cell culture medium supplemented with TGF-β and/or FSH for 24 h (to induce NPPC production) before COCs were added. As shown in Fig. 4d, TGF-β, in the presence of FSH, significantly inhibited oocyte meiotic resumption when COCs were cocultured with MGCs for 1, 2, 3, and 4 h, which may be related to the ability of FSH to enhance TGF-β-induced NPPC expression by the increasing TGFBR2 as well as SMAD proteins (Fig. 3). FSH alone can inhibit oocyte maturation in a short period of time, possibly because FSH can transiently promote cAMP production^40^. TGF-β alone had no effect on oocyte maturation of COCs, possibly because TGF-β could not promote adequate expression of NPPC peptide due to the low levels of TGFBR2 and SMADs. During follicle culture, TGF-β also stimulated the expression of *Nppc* mRNA in MGCs (Fig. S3) and significantly inhibited the meiotic resumption of oocytes within large antral follicles (Fig. 4e). The inhibitory effect of TGF-β on meiotic resumption was blocked by SD208 (Fig. 4d, e). SD208 alone could promote the meiotic resumption of oocytes within the follicles (Fig. 4e), probably because SD208 inhibits TGF-β signaling present in the follicles. Therefore, TGF-β-promoted NPPC in MGCs has an inhibitory effect on oocyte meiotic resumption.

Previous reports indicate that E2 upregulates the expression of *Nppc* mRNA in MGCs and is involved in maintaining meiotic arrest^18,21^. Therefore, we investigated the relationship between TGF-β and E2 on NPPC expression in MGCs. The results showed that TGF-β treatment had no effect on the mRNA expression of ERs (Fig. S4a). In turn, E2 had no effect on the mRNA expression of TGF-β ligands and receptors (Fig. S4b). Moreover, the TGF-β signaling inhibitor SD208 did not inhibit the action of E2 on *Nppc* mRNA expression and oocyte meiotic arrest, and the ER inhibitor ICI182780 did not inhibit the action of TGF-β on *Nppc* mRNA expression and oocyte meiotic arrest (Fig. S4c, d). These results indicate that TGF-β and E2 independently promote NPPC expression in MGCs.

### SMAD3 directly regulates *Nppc* gene transcription

**Fig. 5:**
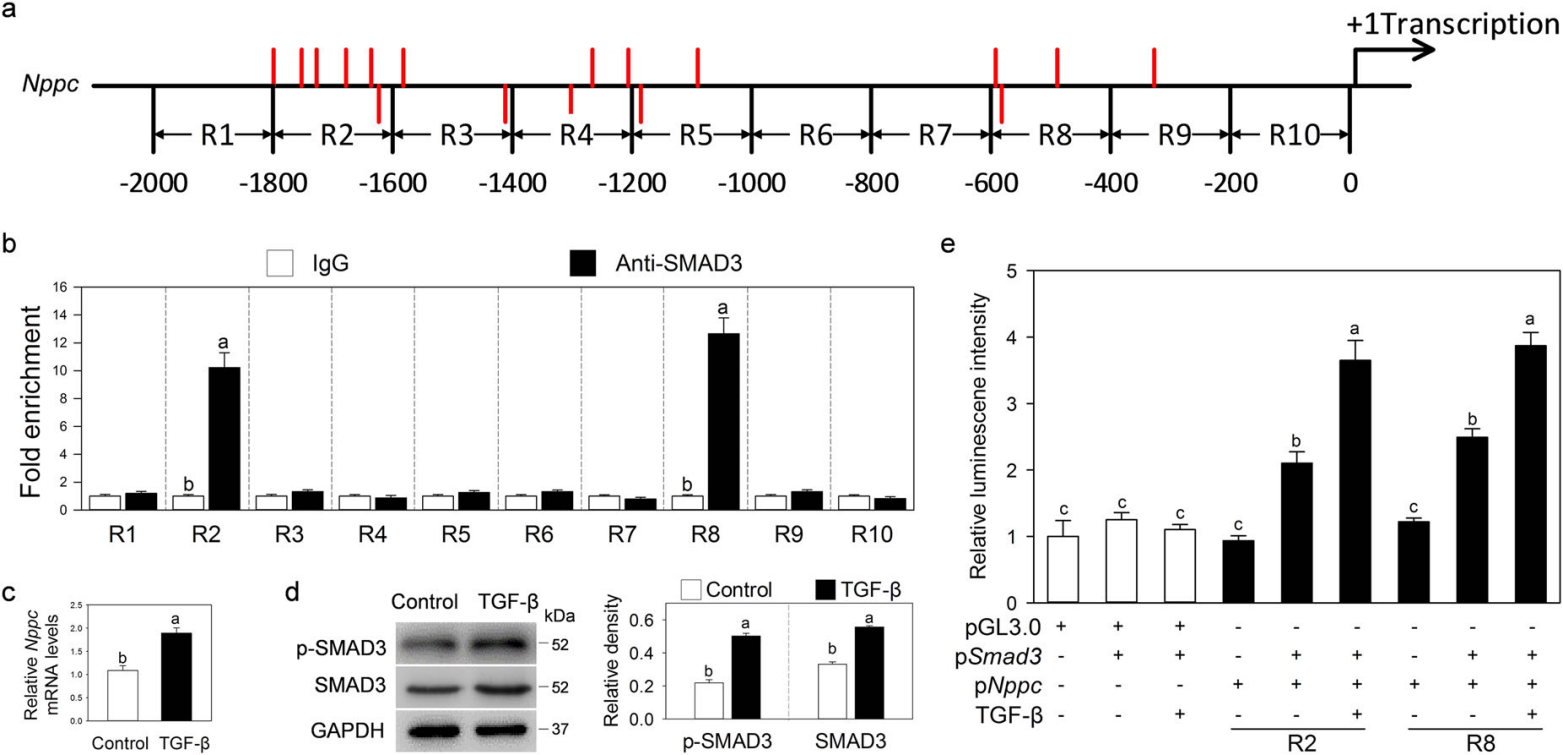
SMAD3 directly regulates *Nppc* gene transcription. **a** Schematic diagram showing the putative SMAD3-binding elements (SBE, AGAC) in the mouse *Nppc* promoter. The *Nppc* promoter sequence was divided into ten regions (designated R1–R10). Each region denotes 200 bp. Red lines represent the potential SMAD3-binding sites in the *Nppc* promoter. The red lines below the axis represent the potential SMAD3-binding sites in the *Nppc* promoter complementary chain. **b** ChIP-qPCR analysis of the interaction between SMAD3 protein and the *Nppc* promoter in MGCs. **c**, **d** Effects of TGF-β on the levels of SMAD3 and phosphorylated SMAD3 (p-SMAD3) protein (**c**) and *Nppc* mRNA (**d**) in KK1 cells. KK1 cells were cultured in cell medium without or with TGF-β (including 10 ng/ml TGFB1, 50 ng/ml TGFB2, and 10 ng/ml TGFB3) for 24 h. The blots shown are representative of three images captured. GAPDH was used as a loading control. **e** Effects of *Smad3* expression vectors (p*Smad3*) on the transiently transfected *Nppc* gene promoter enhancers (R2 and R8) fused to luciferase reporter vectors in KK1 cells. p*Nppc* represents pGL3.0-basic plasmid containing R2 or R8 region of the *Nppc* promoter. KK1 cells were treated without or with TGF-β for 24 h. Bars indicate the mean ± SEM of three independent replicates. Values not indicated by the same letter are significantly different (*P* < 0.05)

After TGF-β stimulation, phosphorylated SMAD2/3 enters the nucleus by binding to SMAD4. In this process, activated SMAD3 binds to SBEs that contain AGAC motifs^29^. We analyzed the *Nppc* promoter 1–2000 base pairs (bp) before the 5′ untranslated region, which was chosen as the core promoter region. Multiple potential SMAD3-binding sites were identified in the *Nppc* promoter (Fig. 5a). Chromatin IP (ChIP) analysis was used to detect whether SMAD3 bound to the *Nppc* promoter to regulate gene transcription. The results showed that SMAD3 bound to the −1800 to −1600 bp (R2) and −600 to −400 bp (R8) regions of the *Nppc* promoter (Fig. 5b).

To further confirm the interaction between SMAD3 protein and the *Nppc* promoter, a mouse granulosa cell line (KK1) responding to TGF-β stimulation was used for a dual-luciferase assay (Fig. 5c, d). We cloned R1–R10 regions and inserted them into the multiple cloning sites of a pGL3.0 vector, respectively. These *Nppc* promoter-driven luciferase reporter plasmids were then transfected with or without a *Smad3* overexpression plasmid into KK1 cells for 24 h. In these assays, SMAD3 significantly enhanced the activities of the R2 and R8 regions, which were further enhanced in response to TGF-β treatment (Fig. 5e and S5). Thus, TGF-β promotes NPPC expression through SMAD3 binding to the *Nppc* promoter.

### Selective *Tgfbr2* depletion in granulosa cells impairs oocyte meiotic arrest, ovulation, and female fertility

**Fig. 6:**
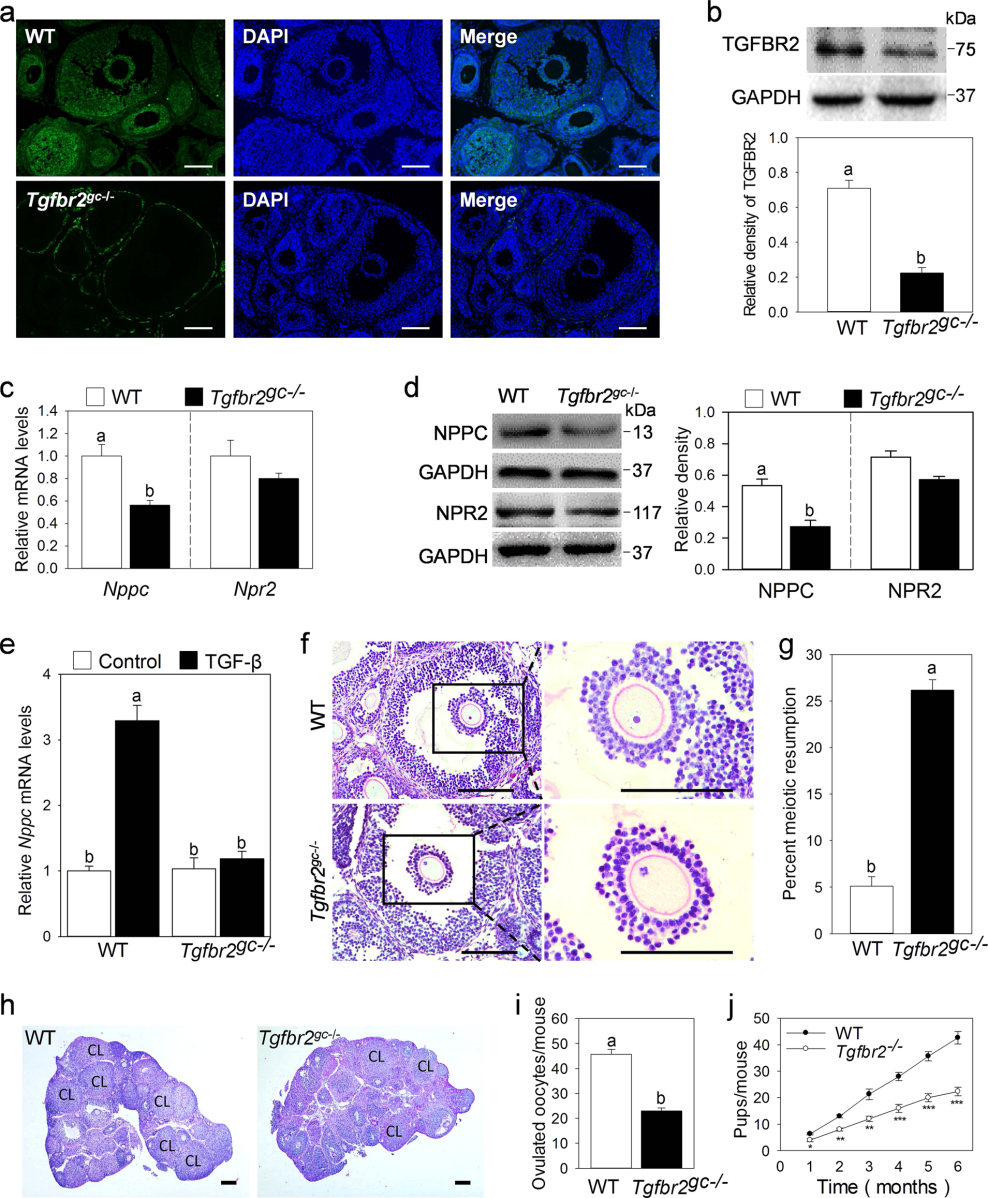
Selective *Tgfbr2* depletion in granulosa cells impairs oocyte meiotic arrest, ovulation, and female fertility. **a, b** The detection of TGFBR2 knockout efficiency in MGCs from eCG-primed mice. The expression levels of TGFBR2 in MGCs were detected by immunofluorescence (**a**) and western blotting (**b**). **c**–**e** Effects of *Tgfbr2* depletion on NPPC and NPR2 expressions. MGCs were isolated from eCG-primed mice. The gene and protein levels of NPPC and NPR2 were detected by qRT-PCR (**c**) and western blotting (**d**), respectively. MGCs isolated from *Tgfbr2*^*gc−/−*^ mice were cultured for 24 h in cell medium without or with TGF-β (including 10 ng/ml TGFB1, 50 ng/ml TGFB2, and 10 ng/ml TGFB3) to detect the effect of TGF-β on *Nppc* mRNA levels (**e**). **f, g** Selective *Tgfbr2* depletion in granulosa cells impairs oocyte meiotic arrest. A prophase-arrested oocyte (GV) within a large antral follicle of a WT ovary and an oocyte with metaphase I (MI) chromosomes within a large antral follicle of a *Tgfbr2*^*gc*−*/−*^ ovary (**f**). Percentages of oocytes that had resumed meiosis, counted in serial sections of ovaries from eCG-primed mice (**g**). The small black boxes indicate the enlarged areas in the following images. *n* = 6. **h**–**j** Selective *Tgfbr2* depletion in granulosa cells impairs ovulation and female fertility. Histology of ovarian sections from WT and *Tgfbr2*^*gc−/−*^ ovaries at 48 h after hCG injection (**h**). CL: corpus luteum. The superovulation assay shows decreased ovulation in *Tgfbr2*^*gc*−*/−*^ mice at 16 h after hCG injection (**i**). Comparison of the cumulative number of progeny per female WT and *Tgfbr2*^*gc−/−*^ female mice over a 6-month period (**j**). *n* = 6. *P ˂ 0.05, **P ˂ 0.01, and ***P ˂ 0.001 compared with corresponding control. The images and blots shown are representative of three images captured in **a**, **b**, **d**, **f**, **h**. Scale bars: 200 μm in **a**, **f**, **h**. GAPDH was used as a loading control in **b**, **d**. Bars indicate the mean ± SEM of three independent replicates. Values not indicated by the same letter are significantly different (*P* < 0.05)

To study the physiological role of TGF-β in NPPC expression and oocyte meiotic arrest, we crossed *Tgfbr2*^*fl/fl*^ mice with *Fshr-Cre* mice to knock out TGF-β-specific type II receptor (TGFBR2) in granulosa cells. In the resulting *Tgfbr2*^*gc*−*/*−^ mice, TGFBR2 expression was dramatically decreased in granulosa cells of antral follicles (Fig. 6a). The reduction in TGFBR2 protein levels in granulosa cells was further confirmed by western blotting results (Fig. 6b). Then, we detected the NPPC level in MGCs. The results showed that the gene and protein levels of NPPC in MGCs from *Tgfbr2*^*gc*−*/−*^ mice were significantly decreased compared with wild-type (WT) controls (Fig. 6c, d). Moreover, TGF-β did not promote the expression of *Nppc* mRNA in the culture of MGCs isolated from *Tgfbr2*^*gc−/−*^ mice (Fig. 6e). *Tgfbr2* depletion slightly decreased the mRNA levels of *Fshr* (encoding FSH receptor) but had no effect on the mRNA levels of *Cyp19a1* (a well-established FSH target gene in granulosa cells) and *Lhcgr* (encoding LH receptor) in MGCs and the levels of E2 in serum (Fig. S6a and b). We next analyzed oocyte meiotic progression by examining serial sections of ovaries from eCG-primed mice. As expected, oocytes in large antral follicles in control ovaries were maintained at the germinal vesicle (GV) stage, and 26% of oocytes in the large antral follicles in *Tgfbr2*^*gc−/−*^ mice had resumed meiosis (Fig. 6f, g). In addition, the number of large antral follicles was significantly reduced in *Tgfbr2*^*gc*−*/−*^ mice (Fig. S6c). In the superovulation test, *Tgfbr2*^*gc−/−*^ mice displayed a 50% ovulation rate compared with WT mice (Fig. 6i). Consistent with the decreased number of ovulations, fewer corpus lutea (CL) were observed in *Tgfbr2*^*gc*−*/−*^ mice at 48 h after hCG treatment (Fig. 6h). A continuous breeding assay demonstrated that *Tgfbr2*^*gc*−*/−*^ female mice had reduced fertility (Fig. 6j). Collectively, our results indicate that TGF-β promotes NPPC expression in MGCs, which is involved in maintaining oocyte meiotic arrest and plays an important role in normal female fertility.

## Discussion

MGCs-secreted NPPC maintains oocyte meiotic arrest by NPR2 producing cGMP^6–8^. In the present study, we found that TGF-β ligands and receptors were mainly expressed in MGCs of mouse ovaries. TGF-β promoted the expression of NPPC in MGCs and inhibited oocyte meiotic resumption in vitro. Moreover, specifically depleting the *Tgfbr2* gene in granulosa cells showed a decrease in NPPC levels in MGCs, resulting in the weak maintenance of oocyte meiosis arrest within large antral follicles. *Tgfbr2* depletion also decreased ovulation number and litter size. Therefore, TGF-β promotes the expression of NPPC in MGCs and participates in maintaining oocyte meiotic arrest.

The immunofluorescence, western blotting, and qRT-PCR analyses revealed that TGFB1, TGFB3, and their receptors were mainly expressed in mouse MGCs, and TGFB2 was primarily expressed in oocytes, which is consistent with previous immunohistochemical results in mouse and other species^41,42^. TGFB1 promotes NPPC expression and secretion in vascular smooth muscle cells^24,25^. In the present study, the addition of TGF-β increased the gene and protein levels of NPPC in the cultured MGCs by SMAD3 binding to the NPPC promoter region and inhibited oocyte meiotic resumption. Furthermore, in *Tgfbr2*^*gc*−*/−*^ mice, the NPPC levels were decreased, and 26% of oocytes resumed meiosis in the large antral follicles. Thus, TGF-β is involved in maintaining oocyte meiotic arrest by promoting the expression of NPPC in MGCs. However, knocking out either *Tgfb1* in granulosa cells or *Tgfb2* in oocytes had no effect on NPPC expression and oocyte maturation (data not shown), indicating that there is a compensatory effect between ligands as reported in mouse embryonic gonadal development^43^. *Tgfbr2* depletion also decreased the number of large antral follicles, ovulation, and little size. According to previous reports, both TGF-β and NPPC could promote follicle development^33,44^. Thus, TGF-β may promote follicle development through the upregulation of NPPC. In *Tgfbr2-*depleted mice, the possible reason for the fertility defect is that the decreased number of large antral follicles, the weakened ability to maintain oocyte meiotic arrest, the reduced oocyte developmental competence^45,46^, and the ovulation defect. E2 upregulates the expression of NPPC in MGCs and maintains oocyte meiotic arrest^18,21^. In our study, TGF-β, independent of the E2 pathway, promotes NPPC expression in MGCs. Knocking out either *Tgfbr2* or ER could only partially cause oocyte precocious meiotic resumption. These results indicate that both TGF-β and E2-promoted NPPC expression in MGCs are required for the maintenance of oocyte meiosis arrest.

The injection of eCG increases NPPC expression in MGCs^6^, but FSH treatment did not increase the NPPC levels in cultured MGCs in our study or another study^18^. This indicates that eCG/FSH promotes NPPC expression indirectly. In vivo, eCG promoted the mRNA and protein levels of TGFB1, TGFBRs, SMAD2, SMAD3, and SMAD4 in MGCs. In vitro, FSH also increased the levels of TGFB1, TGFBR2, SMAD2, SMAD3, and SMAD4, which is consistent with the previous study where FSH upregulated the expression of *Tgfb1* mRNA in cultured caprine follicles^31^. Furthermore, the phosphorylation levels of SMAD3 were also significantly increased by eCG/FSH, suggesting that TGF-β signaling is activated. There was no significant increase in the phosphorylation level of SMAD2, probably due to its lower sensitivity to hormone stimulation during TGF-β signaling transduction^47^. Therefore, the activation of the FSH receptor could promote NPPC expression in MGCs by the upregulation of TGF-β ligands and receptors and TGF-β downstream SMAD proteins to maintain oocyte meiotic arrest during follicular development. However, FSH could not stimulate the expression of NPPC in cultured MGCs. The possible reason is that FSH-induced TGFB1 diffuses into the culture medium, causing a reduction in concentrations to levels unable to act in an autocrine manner. Interestingly, FSH increased TGF-β-promoted NPPC expression in cultured MGCs. This may be because FSH increases the levels of TGFBR2 as well as SMAD proteins. The activation of the LH receptor decreases ovarian NPPC levels and NPR2 activity, resulting in oocyte meiotic resumption^13,15,17^. In a previous study, LH inhibits TGFB1 secretion in theca-interstitial cells^48^. In the present study, hCG treatment significantly decreased the levels of TGFB1, TGFBR2, and SMAD proteins in MGCs, which may result in the decrease in NPPC to induce meiotic resumption.

TGFBR2 was mainly expressed in MGCs. In contrast, bone morphogenetic protein type II receptor (BMPR2), the oocyte-derived paracrine factors (BMP15, growth differentiation factor 9) binding receptor^49^, was mostly expressed in cumulus cells (Fig. S2b). Thus, TGF-β mainly acts on MGCs to promote NPPC expression but had no effect on NPR2 expression. Oocyte-derived paracrine factors act on cumulus cells adjacent to oocytes to promote NPR2 expression^6,50^. This may be the reason for the high levels of NPPC in MGCs and NPR2 in cumulus cells^6^. The depletion of *Smad4* reduces not only NPPC expression but also NPR2 expression in granulosa cells^39^, possibly because both TGF-β and oocyte-derived paracrine factors pathways are blocked in *Smad4* depletion mice.

E2 promotes NPPC/NPR2 expression in MGCs. Here, we show that TGF-β upregulates NPPC expression in MGCs. Both E2 and TGF-β are required for maintaining oocyte meiotic arrest. Moreover, there is a compensatory effect between E2 and TGF-β to ensure normal follicle development and female fertility. Our findings not only contribute to a better understanding of the molecular mechanisms responsible for the regulation of NPPC in the follicles but also provide new therapeutic targets for the treatment of infertility.

## Materials and methods

### Animals and chemicals

WT C57/BL6 female mice and ICR (CD1) female mice were purchased from the Laboratory Animal Center of the Institute of Genetics and Developmental Biology (Beijing, China). *Tgfbr2*^*fl/fl*^ mice (stock number 012603) were purchased from The Jackson Laboratory (Bar Harbor, ME, USA). Mice with granulosa cell-specific knockout of *Tgfbr2* (*Tgfbr2*^*gc−*/−^) were generated by crossing *Tgfbr2*^*fl/fl*^ mice with previously reported *Fshr-Cre* mice^51,52^. Female mice (21–23 days old) were injected with 5 IU of eCG 48 h before use to stimulate follicle development. In some experiments, the female mice were treated with 5 IU eCG followed by 5 IU hCG to stimulate ovulation. All animal procedures were approved by the Institutional Animal Care and Use Committee of China Agricultural University and maintained according to the Guide for the Care and Use of Laboratory Animals (Institute for Learning and Animal Research at China Agricultural University). The reagents used in this study, unless otherwise stated, were purchased from Sigma-Aldrich (St. Louis, MO, USA).

### Isolation of MGCs, cumulus cells, and oocytes

MGCs were collected through gentle puncture of antral follicles from 21–23 days old mice with a 25-gauge syringe needle. In some experiments, MGCs were collected from eCG-primed mice or eCG-primed mice followed by 5 IU hCG. COCs with the oocytes at the GV stage were collected from eCG-primed mice, and the cumulus cells were stripped from the oocytes by repeatedly drawing the oocytes in and out of a glass pipet slightly smaller in diameter than the oocytes. The collected MGCs, cumulus cells and oocytes were immediately frozen in liquid nitrogen and stored at −80 °C until analysis for gene or protein levels.

### MGCs and COCs cultures

MGCs isolated from 21–23 days old mice were washed and then cultured at a density of 1 × 10^6^ cells. The cell culture medium was Dulbecco’s Modified Eagle Medium (DMEM)/F12 with 2.2 mg/ml NaHCO_3_ and 5% fetal bovine serum (FBS) (Thermo Fisher Scientific, Waltham, MA, USA). The culture was carried out at 37 °C in a controlled atmosphere of 5% O_2_, 5% CO_2_, and 90% N_2_. After the overnight culture, the unattached, nonviable MGCs were then removed by washing with this medium. The attached cells were then cultured with FSH (10 ng/ml), E2 (100 nmol/l), TGFB1 (10 ng/ml), TGFB2 (50 ng/ml), TGFB3 (10 ng/ml), and/or SD208 (1 μmol/l) for 24 h. Then, the MGCs were either collected using trypsin-EDTA (0.25%) digestion for gene expression and protein analysis or continuously cocultured with COCs for varying times. At the end of culture, oocyte meiotic resumption was assessed by scoring released oocytes for GV breakdown (GVB). GVB was scored by the absence of an obvious GV in the oocyte.

### Isolation and culture of follicles

Large antral follicles (350–400 μm) from eCG-primed mice were separated by gentle dissection with a 25-gauge syringe needle. The follicle culture medium was bicarbonate-buffered minimum Eagle medium (MEM)-alpha with Earle balanced salts supplemented with 2.5 mg/ml insulin, 5 µg/ml transferrin, 75 µg/ml penicillin G, 50 µg/ml streptomycin sulfate, 0.23 mM pyruvate, and 3 mg/ml bovine serum albumin. The follicles were cultured on Millicell culture inserts (Millipore, Billerica, MA, USA) supplemented with SD208 (1 μmol/l), ICI182780 (10 μmol/l), E2, TGFB1, TGFB2, and/or TGFB3. The culture was carried out at 37 °C in a controlled atmosphere (5% O_2_, 5% CO_2_, and 90% N_2_) for 4 h. At the end of culture, the MGCs and oocytes were isolated from the follicles for the analysis of gene expression and meiotic resumption, respectively.

### Immunofluorescence and histologic analysis

For immunofluorescence, the ovaries from eCG-primed mice were fixed with 4% paraformaldehyde for 12 h, dehydrated, embedded in paraffin and sectioned at 5 μm. The sections were deparaffinized and rehydrated through a graded ethanol series and subjected to antigen retrieval using 0.01% sodium citrate buffer (pH 6.0). The nonspecific binding was blocked with 10% normal donkey serum for 1 h at room temperature. Then, the sections were incubated with primary antibodies overnight at 4 °C, followed by Alexa Fluor 488-conjugated secondary antibodies (1:100, Thermo Fisher Scientific, Waltham, MA, USA) at room temperature for 2 h. Subsequently, the sections were washed with PBS and stained with DAPI for 5 min. The primary antibodies used were as follows: rabbit anti-TGFB1 (ab92486; Abcam, Cambridge, UK) at 1:200, mouse anti-TGFB2 (ab36495; Abcam) at 1:50, rabbit anti-TGFB3 (ab15537; Abcam) at 1:50, rabbit anti-TGFBR1 (ab31013; Abcam) at 1:100, rabbit anti-TGFBR2 (ab186838; Abcam) at 1:200, rabbit anti-SMAD2/3 (8685; Cell Signaling Technology, Danvers, MA, USA) at 1:100, rabbit anti-SMAD4 (46535; Cell Signaling Technology) at 1:100, and rabbit anti-phospho-SMAD3 (9520; Cell Signaling Technology) at 1:100. In some experiments, the cultured MGCs were fixed with 4% paraformaldehyde for 20 min, permeabilized with PBS containing 0.3% Triton X-100 (PBST) for 30 min, and blocked with 5% BSA in PBST at room temperature. Then, cells were used for immunofluorescence staining with the antibodies as described above.

For histologic analysis, paraffin-embedded ovarian samples prepared from eCG-primed mice or eCG-primed mice followed by 5 IU hCG were serially sectioned at 5 μm and stained with periodic acid/Schiff reagent and hematoxylin. The number of large antral follicles and oocytes with meiotic resumption was counted by examining serial sections through the entire ovary. The oocyte count method was determined as previously reported^6^. If the GV was no longer present and condensed chromosomes were visible, oocytes were scored as having resumed meiosis.

### Western blotting

Total proteins were extracted in a tissue and cell lysis solution (CellChip Biotechnology, Beijing, China), and protein concentration was quantified using the BCA Protein Assay Kit (Beyotime, Shanghai, China). Then, the protein lysates (30 μg total protein per lane) were separated by sodium dodecyl sulfate-polyacrylamide gel electrophoresis and electrically transferred to polyvinylidene fluoride membranes (Millipore). Membranes were first blocked with 5% nonfat milk in Tris-buffered saline (pH = 7.6) for 2 h at room temperature, followed by incubation overnight at 4 °C with primary antibodies (each diluted 1:1000): TGFB1, TGFB2, TGFB3, TGFBR1, TGFBR2, SMAD2/3, SMAD4, phospho-SMAD3, rabbit anti-SMAD3 (9523; Cell Signaling Technology), rabbit anti-phospho-SMAD2/3 (8828; Cell Signaling Technology), rabbit anti-CNP (sc-374043; Santa Cruz Biotechnology, CA, USA. It can be used to detect CNP precursor and all active peptides by western blotting), rabbit anti-NPR2 (ab14357; Abcam), or rabbit anti-GAPDH (5174, Cell Signaling Technology). Then, the membranes were washed and further incubated for 1 h at room temperature with horseradish peroxidase-conjugated secondary antibodies (each diluted 1:5000) (Zhongshan Golden Bridge Biotechnology, Beijing, China). Protein visualization was performed with a Tanon 5200 chemiluminescent imaging system (Tanon, Shanghai, China). The levels of GAPDH were detected as a loading control.

### RNA extraction and quantitative RT-PCR analysis

Total RNA was extracted and purified from cells using the RNeasy micro-RNA Isolation Kit (Qiagen, Valencia, CA, USA), and reverse transcription into cDNA was conducted using the QuantiTect Reverse Transcription System (Qiagen) according to the manufacturer’s instructions. The relative amount of target gene expression for each sample was determined using an ABI 7500 real-time PCR instrument (Applied Biosystems, Foster City, CA, USA). The relative gene expression was quantified on the basis of the threshold cycle value and normalized using a housekeeping gene, ribosomal protein L19 (*Rpl19*). The relative transcript level of the control group was set as 1, and relative transcript levels of other samples were compared with the control. qRT-PCR primers for *Nppc*, *Npr2*, and *Rpl19* were reported previously^5^. Other primer sequences used for qRT-PCR are shown in Supplementary Table S1.

### Measurement of NPPC levels

The cultured MGCs with different treatments were collected and transferred to a 1.5 ml centrifuge tube. Then, the samples were treated according to a previous study^53^. Briefly, MGCs were boiled in 100 μl of 1.0 M acetic acid for 5 min and homogenized on ice using a tissue homogenizer (T10 basic, IKA, Germany) and were lysed with an ultrasonic cell pulverizer (Scientz-IID, Ningbo, China). We added 500 μl of MeOH to dissolve the lipids in the sample. After centrifugation at 20,000 × *g* at 4 °C for 30 min, the supernatant containing 2.0–5.0 mg of protein was frozen in liquid nitrogen. Lyophilized sample extracts were assayed for NPPC levels using a luciferase immunoassay kit (Phoenix Pharmaceuticals, Belmont, CA, USA) according to the manufacturer’s instructions.

### Chromatin IP (ChIP) assay

ChIP assays were performed using a ChIP kit (Active Motif, Carlsbad, CA, USA) according to the manufacturer’s instructions. The MGCs harvested from 21–23 days old mice were washed three times with ice-cold PBS. Samples were fixed, cross-linked and lysed. The lysate was centrifuged and resuspended in 350 μl of digestion buffer. Then, chromatin was enzymatically sheared until the average DNA length was ~200–300 bp, as evaluated by agarose gel electrophoresis. The sample containing 25 μg of sheared chromatin was used for immunoprecipitation with 3 μg of SMAD3 antibody or normal rabbit IgG (sc-2774; Santa Cruz Biotechnology, Santa Cruz, CA, USA). Following elution, cross-link reversal and proteinase K digestion, the DNA fragments were purified by phenol/chloroform extraction and ethanol precipitation from the protein/DNA complexes. Immunoprecipitated chromatin fragments and input chromatin were detected by qRT-PCR. The primers used for the ChIP assay are indicated in Supplementary Table S2.

### Plasmids constructs and a dual-luciferase reporter assay

For the luciferase assay, the DNA fragments containing the R1–R10 regions of the NPPC promoter (according to the results of the ChIP assay) were amplified by PCR from mouse genomic DNA using specific primers and inserted into the pGL3.0 luciferase reporter vector (E1910; Promega, Madison, WI, USA). The forward primer contained a restriction enzyme site of MluI, and the reverse primer contained a restriction enzyme site of XhoI. The cDNA-derived mouse *Smad3* sequence was amplified using PCR with the primer containing the MluI–KpnI restriction sites and inserted into the pcDNA3.1 vector (1332; Addgene, Cambridge, MA, USA). Primer sequences were listed in Supplementary Table S3. KK1 cells were maintained in cell culture medium at 37 °C with 5% CO_2_. This cells were transiently transfected with *Smad3* expression vector, *Nppc* luciferase reporter vector, and pTK-Renilla vector using Lipofectamine 2000 reagent (Thermo Fisher Scientific). An empty luciferase reporter vector was used as a control. After 24 h of transfection, the cells were harvested, and luciferase activities were measured using the Dual-Luc Assay Kit (E1960; Promega). Values shown by the fluc to rluc ratio were normalized to an empty luciferase reporter control.

### E2 level assays

The 21–23 days old mice were placed in the supine position, and blood was collected by cardiac puncture. Serum and blood cells were separated by centrifugation, and the serum E2 level was determined by Beijing North Biotechnology Institute (Beijing, China).

### Superovulation and fertility

To investigate ovulation, 21–23 days old female *Tgfbr2*^*gc*−/−^ mice and WT female mice were injected with 5 IU eCG followed by 5 IU hCG 48 h later to induce ovulation. Then, we counted the number of oocytes by separating COCs from the ampulla at 16 h after hCG treatment. To evaluate reproductive performance, 6-week-old *Tgfbr2*^*gc*−/−^ female mice and WT female mice were continually mated to WT males of known fertility. The number of litters was recorded over a 6-month period.

### Statistical analysis

All experiments were repeated at least three times, and values were expressed as the mean ± SEM. The proportional data were subjected to an arcsine transformation. A *t*-test was used to analyze the significant difference between the treatment and control groups. ANOVA (SAS Institute, Inc., Cary, NC, USA) was used to compare data from multiple groups. When a significant F ratio was detected by the ANOVA, the groups were compared using the Holm–Šidák test. Values of *P* < 0.05 were considered significant.

## Supplementary information

Figure S1

Figure S2

Figure S3

Figure S4

Figure S5

Figure S6

Supplemental Material
