# Supplementary figures and images for: Transforming growth factor-β is involved in maintaining oocyte meiotic arrest by promoting natriuretic peptide type C expression in mouse granulosa cells
Source: Cell Death Dis. 2019 Jul 22;10(8):558. doi: 10.1038/s41419-019-1797-5 (PMC6646305; doi:10.1038/s41419-019-1797-5)

Fig. 1c

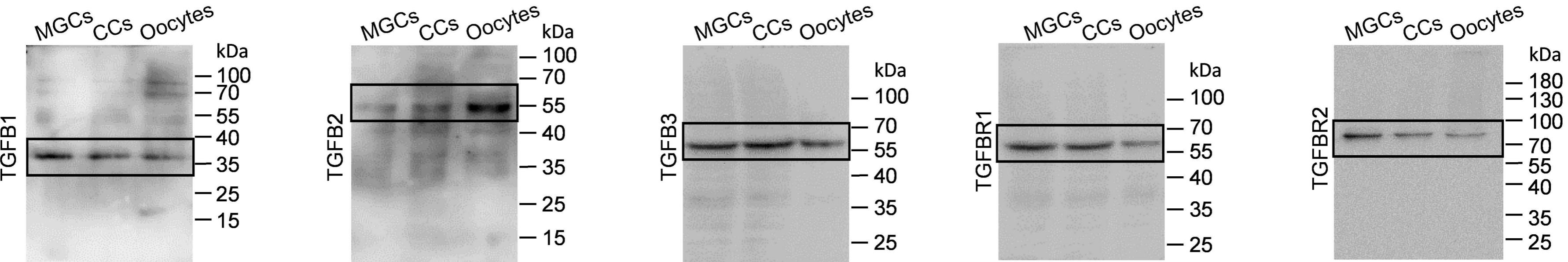

Fig. 4b

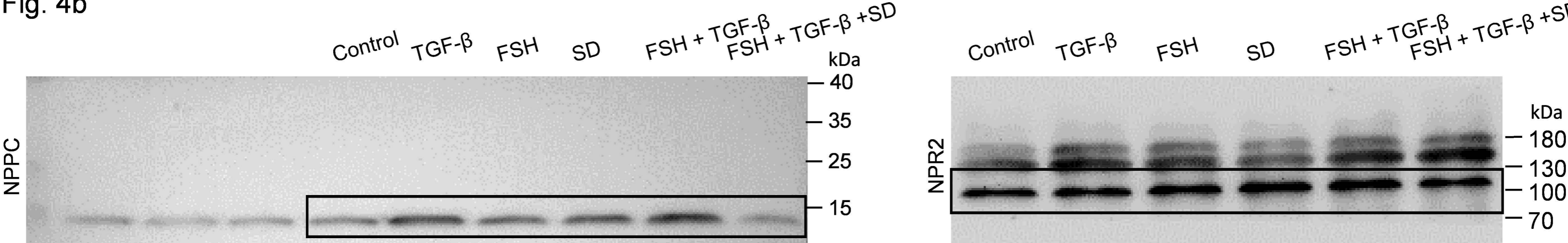

Supplement: Supplementary file 1 — Figure S1 [file 41419_2019_1797_MOESM1_ESM.pdf]

**a**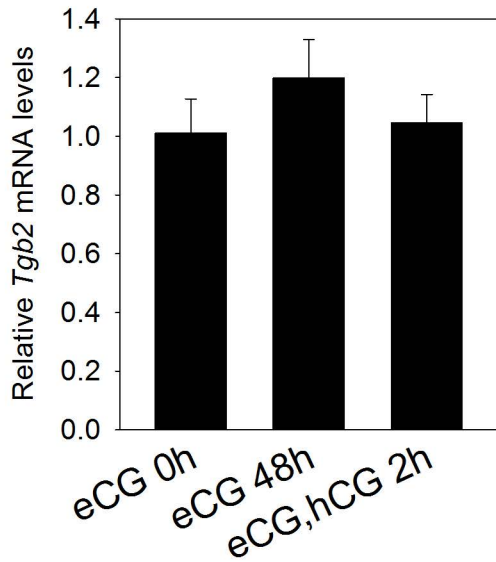**b**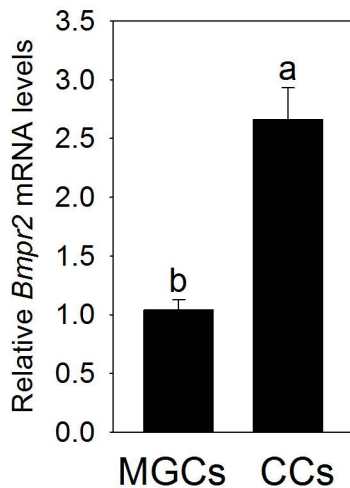

Supplement: Supplementary file 2 — Figure S2 [file 41419_2019_1797_MOESM2_ESM.pdf]

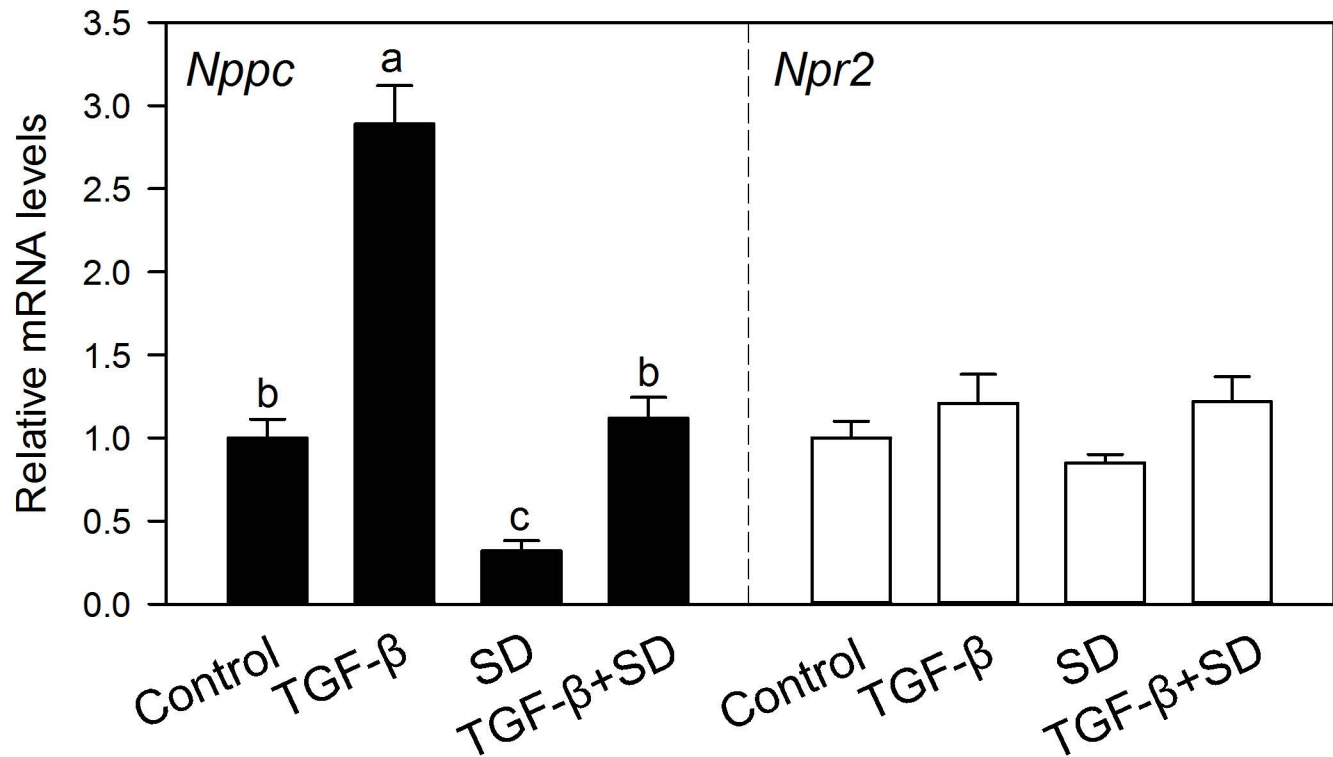

Supplement: Supplementary file 3 — Figure S3 [file 41419_2019_1797_MOESM3_ESM.pdf]

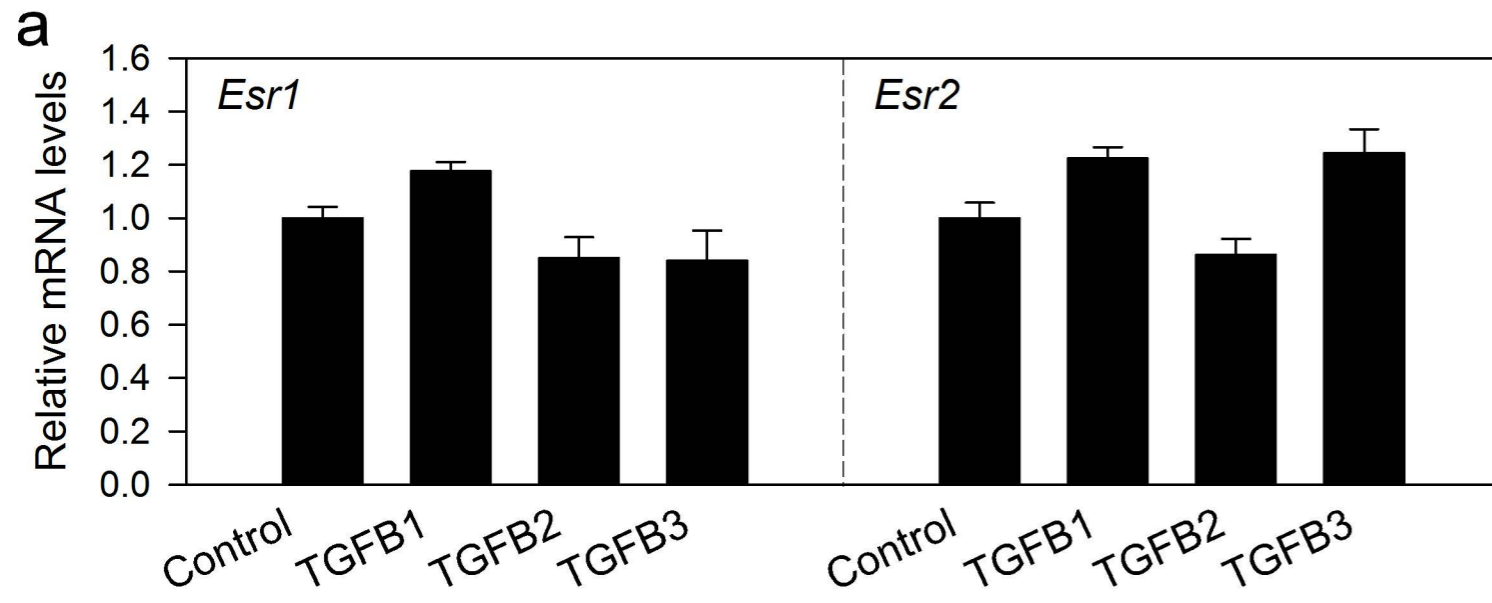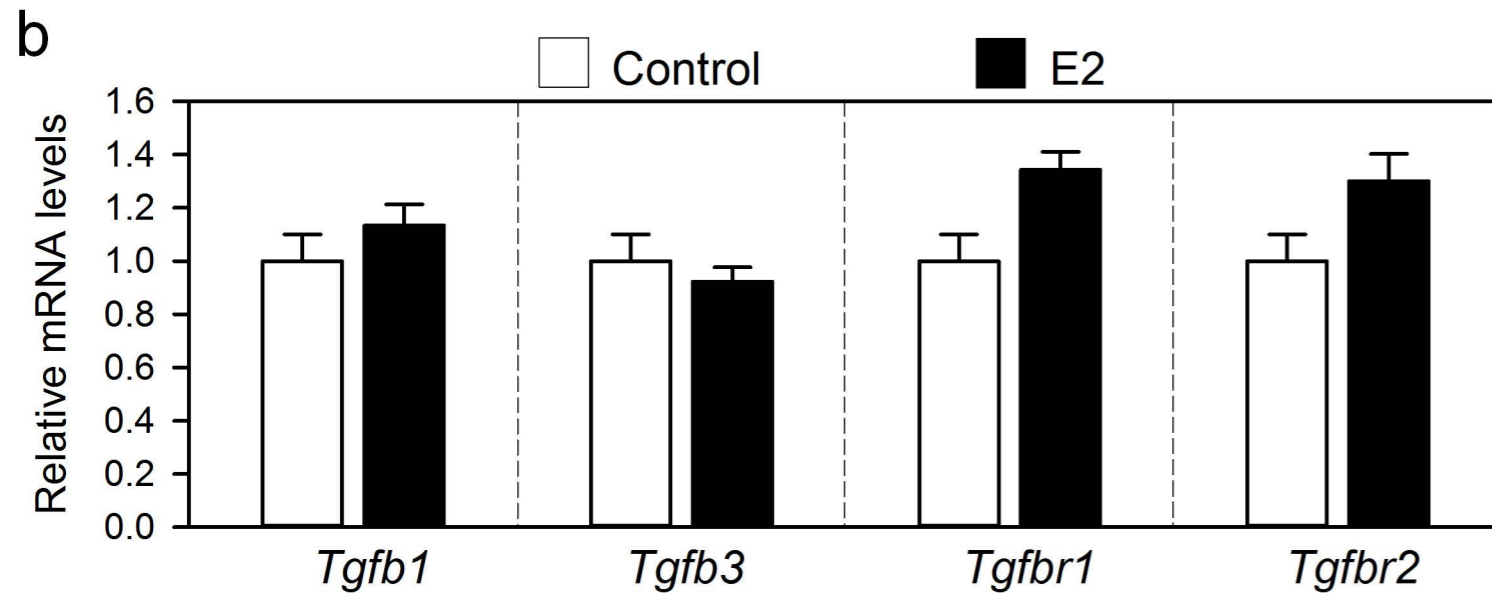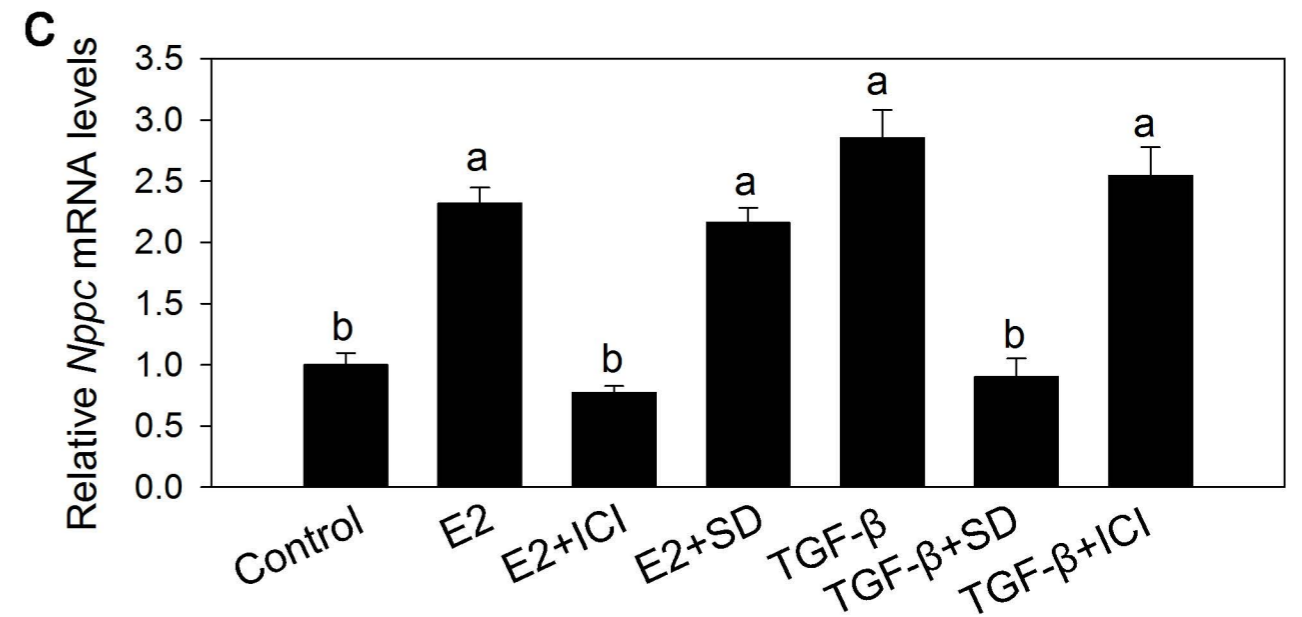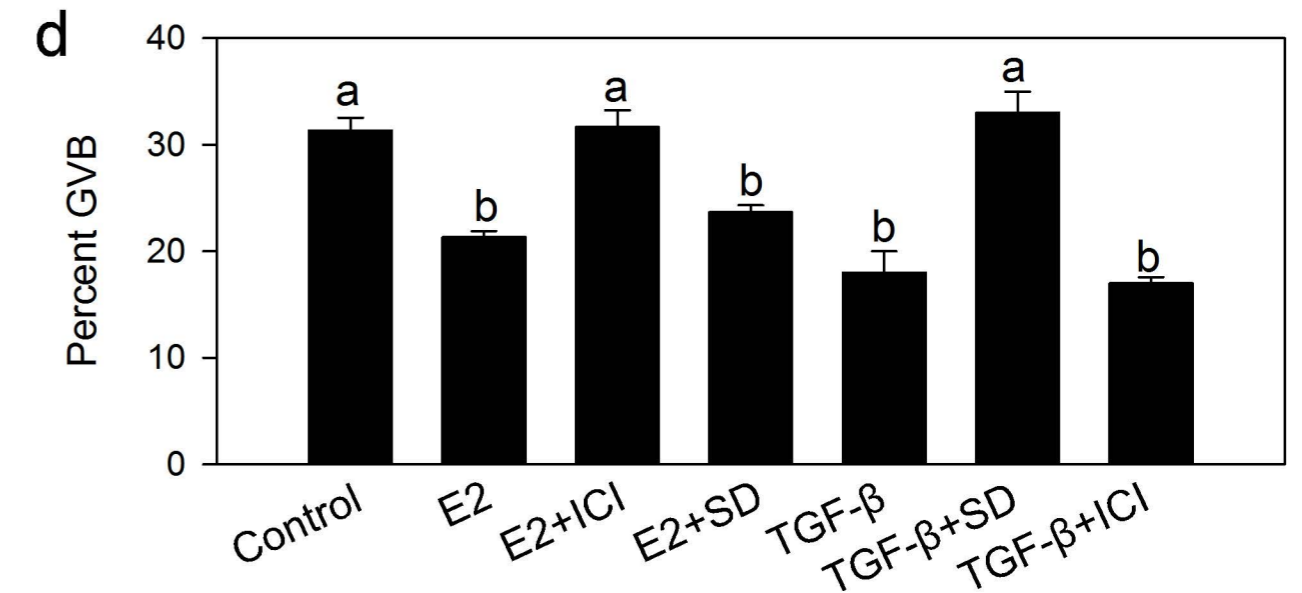

Supplement: Supplementary file 4 — Figure S4 [file 41419_2019_1797_MOESM4_ESM.pdf]

Relative luminescence intensity

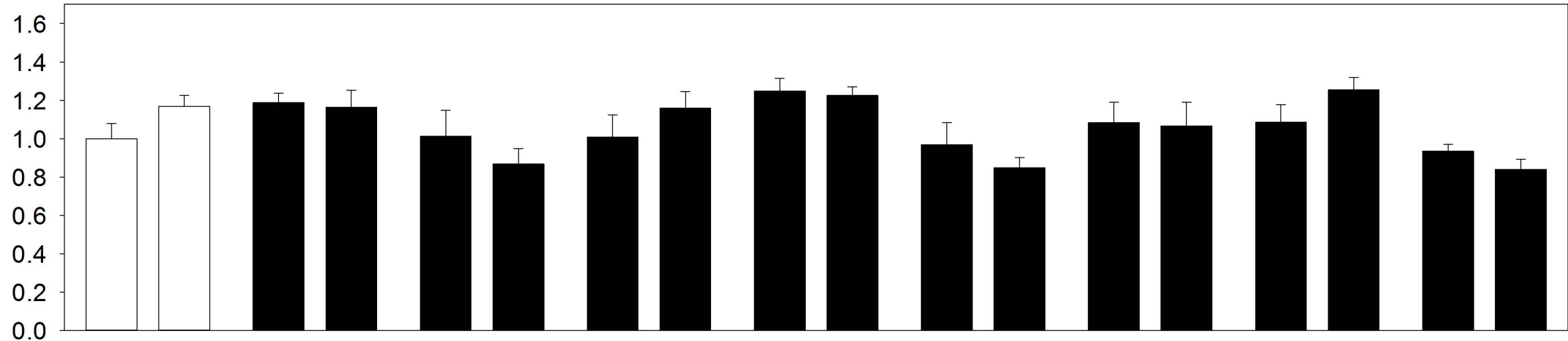

|        |   |   |       |   |       |   |       |   |       |   |       |   |       |   |       |   |       |  |
|--------|---|---|-------|---|-------|---|-------|---|-------|---|-------|---|-------|---|-------|---|-------|--|
| pGL3.0 | + | + | -     | - | -     | - | -     | - | -     | - | -     | - | -     | - | -     | - | -     |  |
| pSmad3 | - | + | -     | + | -     | + | -     | + | -     | + | -     | + | -     | + | -     | + | +     |  |
| pNppc  | - | - | +     | + | +     | + | +     | + | +     | + | +     | + | +     | + | +     | + | +     |  |
|        |   |   | <hr/> |  |
|        |   |   | R1    |   | R3    |   | R4    |   | R5    |   | R6    |   | R7    |   | R9    |   | R10   |  |

Supplement: Supplementary file 5 — Figure S5 [file 41419_2019_1797_MOESM5_ESM.pdf]

**a**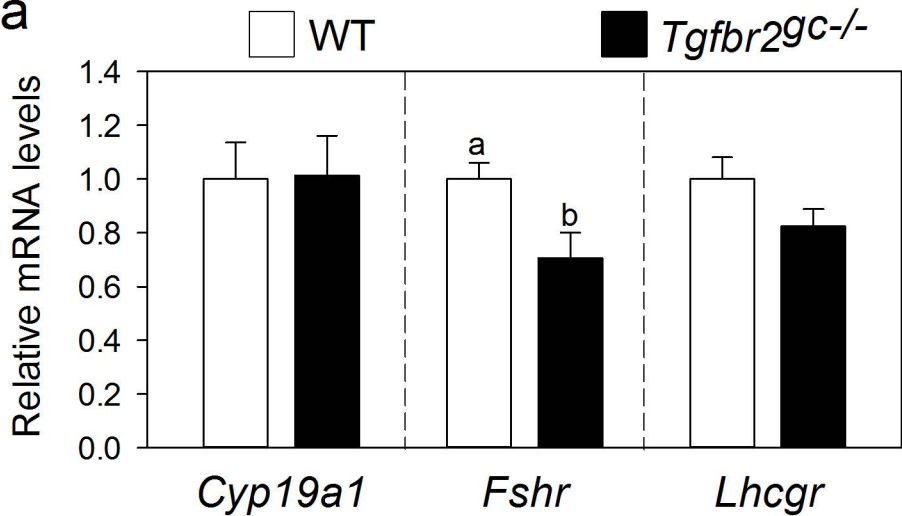**b**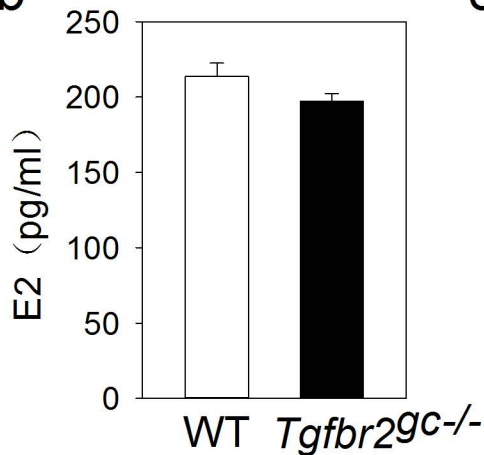**c**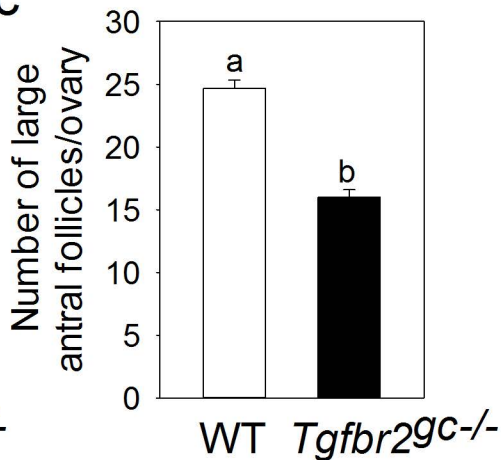

Supplement: Supplementary file 6 — Figure S6 [file 41419_2019_1797_MOESM6_ESM.pdf]
