# Supplementary material for: Transforming growth factor-β is involved in maintaining oocyte meiotic arrest by promoting natriuretic peptide type C expression in mouse granulosa cells
Source: Cell Death Dis. 2019 Jul 22;10(8):558. doi: 10.1038/s41419-019-1797-5 (PMC6646305; doi:10.1038/s41419-019-1797-5)
Supplement: Supplementary file 7 — Supplemental Material [file 41419_2019_1797_MOESM7_ESM.docx]

**Supplemental Material**

Jing Yang, et al.

**Transforming growth factor-β is involved in maintaining oocyte meiotic arrest by promoting natriuretic peptide type C expression in mouse granulosa cells**

**Supplementary Figure Legends**

**Fig. S1 Uncropped scans of the most important Western blotting results**. Black boxes highlight the lanes used in the figures.

**Fig. S2 The mRNA expression of *Tgfb2* in oocytes** **and *Bmpr2* in MGCs and cumulus cells.** (a) Quantitative RT-PCR analysis of the gene expression of *Tgfb2* in oocytes. (b) Comparison of steady-state levels of *Bmpr2* mRNA in MGCs and cumulus cells (CCs) isolated from eCG-primed mice. Bars indicate the mean ± SEM of three experiments. Values not indicated by the same letter are significantly different (*P* < 0.05).

**Fig. S3 Effect of TGF-β on *Nppc* mRNA levels in MGCs.** Large antral follicles isolated from eCG-primed mice were cultured in the medium, supplemented with TGF-β (including 10 ng/ml TGFB1, 50 ng/ml TGFB2, 10 ng/ml TGFB3), and/or 1 μmol/l SD208 (SD) for 4 h, and then MGCs were collected for gene analysis. Bars indicate the mean ± SEM of three experiments. *n* = 30 for each treatment across all three replicates. Values not indicated by the same letter are significantly different (*P* < 0.05).

**Fig. S4 Relationship between TGF-β and E2 in regulating *Nppc* mRNA levels and oocyte meiotic resumption**. (a) Effects of TGFB1, TGFB2 and TGFB3 on *Esr1* and *Esr2* mRNA levels in MGCs. MGCs were collected from prepubertal mice and cultured for 24 h in cell culture medium supplemented with 10 ng/ml TGFB1, 50 ng/ml TGFB2, or 10 ng/ml TGFB3. *Esr1* and *Esr2* are the corresponding gene names of ERα and ERβ. (b) Effects of E2 on *Tgfb1, Tgfb3, Tgfbr1* and *Tgfbr2* mRNA levels in MGCs. MGCs were collected from prepubertal mice and cultured for 24 h in cell medium without or with 100 nmol/l E2. (c and d) Effects of ICI182780 (ICI) and SD208 (SD) on *Nppc* mRNA levels and oocyte meiotic resumption. Large antral follicles isolated from eCG-primed mice were cultured in follicle culture medium supplemented with 100 nmol/l E2, 10 μmol/l ICI182780, TGF-β (including 10 ng/ml TGFB1, 50 ng/ml TGFB2, 10 ng/ml TGFB3), and/or 1 μmol/l SD208 for 4 h. At the end of culture, MGCs were collected from follicles for gene analysis (c), and the proportion of oocytes having undergone GVB was counted at the same time (d). Bars indicate the mean ± SEM of three experiments. *n* = 30 for each treatment across all three replicates. Values not indicated by the same letter are significantly different (*P* < 0.05).

**Fig. S5 Effects of Smad3 expression vectors (pSmad3) on the transiently transfected Nppc gene promoters fused to luciferase reporter vectors were assayed.** pNppc represents pGL3.0-basic plasmid containing 8 regions of the *Nppc* promoter. Bars indicate the mean ± SEM of three independent replicates.

**Fig. S6 The effects of *Tgfbr2* depletion on *Cyp19a1*, *Fshr*, and *Lhcgr* mRNA, serum E2 concentration and follicle development**. MGCs and serum were collected from eCG-primed mice to analyze the gene expression of *Cyp19a1*, *Fshr*, and *Lhcgr* (a), and the E2 concentration (b), respectively. The number of large antral follicles was counted by examining serial sections through the entire ovary of eCG-primed mice (c). Bars indicate the mean ± SEM of three experiments. Values not indicated by the same letter are significantly different (*P* < 0.05).

**Supplementary Tables**

**Table S1: Primers for RT-qPCR**

| **Genes** | **Forward primer (5’-3’)** | **Reverse primer (5’-3’)** |
| --- | --- | --- |
| *Tgfb1* | CTCCCGTGGCTTCTAGTGC | GCCTTAGTTTGGACAGGATCTG |
| *Tgfb2* | CTTCGACGTGACAGACGCT | GCAGGGGCAGTGTAAACTTATT |
| *Tgfb3* | CCTGGCCCTGCTGAACTTG | TTGATGTGGCCGAAGTCCAAC |
| *Tgfbr1* | TCTGCATTGCACTTATGCTGA | AAAGGGCGATCTAGTGATGGA |
| *Tgfbr2* | TTGGATTGCCAGTGCTAACCC | AACAAGCCACAGTAACATGACA |
| *Esr1* | CCTCCCGCCTTCTACAGGT | CACACGGCACAGTAGCGAG |
| *Esr2* | CTGTGCCTCTTCTCACAAGGA | TGCTCCAAGGGTAGGATGGAC |
| *Lhcgr* | CGCCCGACTATCTCTCACCTA | GACAGATTGAGGAGGTTGTCAAA |
| *Fshr* | CCTTGCTCCTGGTCTCCTTG | CTCGGTCACCTTGCTATCTTG |
| *Cyp19a1* | ATGTTCTTGGAAATGCTGAACCC | AGGACCTGGTATTGAAGACGAG |

**Table S2: Primers for the ChIP assay**

| **Genes** | **Forward primer (5’-3’)** | **Reverse primer (5’-3’)** |
| --- | --- | --- |
| *Nppc-R1* | ATGGCCTATTGAGGCTTGGA | TTCAGTGATGTACTGGCCCTTC |
| *Nppc-R2* | TTCAGGAAGGGCCAGTACATC | TCCTGTCTTGGTAATGGGCTG |
| *Nppc-R3* | ACCAGCCCATTACCAAGACAG | ATCCAAGCTTTGAGACCGCA |
| *Nppc-R4* | TGGTACTCTCCTGCCCCTTA | GTTCTGGAAGGTTGGTGGCT |
| *Nppc-R5* | ATGGAGATGAGCCACCAACC | GTGCCGTCTACTTCCTCGTC |
| *Nppc-R6* | ACGGCACACTCAGTACACTC | CAGGCTTGAGCGAGGGAAAT |
| *Nppc-R7* | GGGACCCTGCCAACATTCAT | TCTCGGATACCCACGAGTGA |
| *Nppc-R8* | AACCCCAAACTCAATGGCGA | GTGCGGCCCAAAATCTACAC |
| *Nppc-R9* | GGCACGGGAAGAGCAATGGG | CTGTCGGAGAAAAGAGTGGA |
| *Nppc-R10* | GTCCCGAGAACCCCGCCAGG | CTCGTGCAGAAGGCGGGCCC |

**Table S3: Primers for plasmid construction**

| **Genes** | **Forward primer (5’-3’)** | **Reverse primer (5’-3’)** |
| --- | --- | --- |
| *pNppc-R1* | ACGCGTTGGTACTCTCCTGCCCCTTA | CTCGAGGTTCTGGAAGGTTGGTGGCT |
| *pNppc-R2* | GACGCGTGCTATCAAGACTGAACAGTCCCA | CCTCGAGGCCTAAGGGGCAGGAGAGTA |
| *pNppc-R3* | ACGCGTACCAGCCCATTACCAAGACAG | CCTCGAGATCCAAGCTTTGAGACCGCA |
| *pNppc-R4* | GACGCGTTGGTACTCTCCTGCCCCTTA | CCTCGAGGTTCTGGAAGGTTGGTGGCT |
| *pNppc-R5* | ACGCGTATGGAGATGAGCCACCAACC | CTCGAGGTGCCGTCTACTTCCTCGTC |
| *pNppc-R6* | ACGCGTCACGGCACACTCAGTACACTC | CTCGAGCAGGCTTGAGCGAGGGAAAT |
| *pNppc-R7* | ACGCGTGGGACCCTGCCAACATTCAT | CTCGAGTCTCGGATACCCACGAGTGA |
| *pNppc-R8* | GACGCGTACCTTCCAGAACTGACAAAGG | CCTCGAGACTGAGTGTGCCGTCTACTT |
| *pNppc-R9* | ACGCGTCGGCACGGGAAGAGCAATGGG | CTCGAGGCTGTCGGAGAAAAGAGTGGA |
| *pNppc-R10* | GACGCGTGTCCCGAGAACCCCGCCAGG | CCTCGAGCTCGTGCAGAAGGCGGGCCC |
| *pSmad3* | CGGGATCCATGTCGCCATCCTGCCCTT | CCGCTCGAGCTAAGACACTGGAACAGC |
